# Responses of coastal fishery resources to rapid environmental changes

**DOI:** 10.1111/jfb.15138

**Published:** 2022-07-07

**Authors:** Heikki Peltonen, Benjamin Weigel

**Affiliations:** ^1^ Finnish Environment Institute (SYKE) Helsinki Finland; ^2^ Research Centre for Ecological Change, Organismal and Evolutionary Biology Research Programme, Faculty of Biological and Environmental Sciences University of Helsinki Helsinki Finland

**Keywords:** Baltic Sea, climate change, fish community, fishery yield, joint species distribution model

## Abstract

Coastal systems experience strong impacts of ongoing environmental change, affecting fish communities and subsequently fishery yields. In the Baltic Sea, the combined effects of climate‐induced changes and eutrophication‐related pressures constitute major threats to its living resources. Although much work has been devoted to uncovering environmental impacts on the commercially most valuable fish stocks, only little is known about community‐wide responses of fished species and how environmental change may affect their yield. In this study, the authors use a joint species distribution modelling framework to disentangle environmental impacts on species‐specific fishery yields of 16 fished species along the coast of Finland over four decades. The authors show that environmental covariates substantially contributed to variations in fishery yields and are likely to have strong impacts on fished resources also in the future. Salinity and near‐bottom oxygen concentration emerged as the strongest environmental drivers of yields at the community level, whereas temperature was particularly important for cod (*Gadus morhua*) and sprat (*Sprattus sprattus*) yields. The authors found shore density to be an important predictor for fisheries resources especially for freshwater fish. The results of this study suggest that the changes in environmental conditions during the past four decades had a positive effect on the yields of freshwater and warm‐affinity species, whereas yields of marine cold‐affinity species have been mainly negatively affected by contracting favourable habitats, becoming warmer and less saline.

## 1 INTRODUCTION

The Baltic Sea has undergone significant changes in its major fish stocks because of environmental change during the past decades (Köster *et al*., 2003; Mackenzie *et al*., 2007). Climate‐induced changes, affecting, *e.g.*, temperature and salinity, in concert with eutrophication are considered among the most important drivers of this change (Mackenzie *et al*., 2007; Möllmann *et al*., 2009; Reusch *et al*., 2018). Nonetheless, fisheries and fisheries management have also contributed to the population dynamics of fish (Aps & Lassen, 2010). Although the main body of research centres on changes and dynamics of the commercially most relevant fish stocks such as cod (*Gadus morhua* L.), herring (*Clupea harengus* L.) and sprat (*Sprattus sprattus* L.), only limited information is available on more detailed community‐wide responses of fished species and their yields (Mustamäki & Mattila, 2015; Olsson *et al*., 2012; Uusitalo *et al*., 2012). Especially coastal areas harbour species‐rich communities with large numbers of fishes, also from pelagic regions, using this area during one or several of their life stages (Kraufvelin *et al*., 2018; Seitz *et al*., 2014). To improve ecosystem‐based management, it is desirable to understand how community dynamics of fisheries yields are shaped by anthropogenic pressure, considering these communities as the multispecies complex they are. Therefore, disentangling the environmental drivers that contribute to the spatiotemporal progression of fished yields is needed to anticipate possible future developments under continued global change.

During the past decade, species distribution modelling has become a common and fundamental tool to understand and predict environmental filtering of species communities (Warton *et al*., 2015). Although most of the existing literature and tools deal with single‐species modelling approaches, the need for multispecies approaches, *i.e.*, joint species distribution models (JSDM), has been emerging to better understand community‐level responses to altered environments (Ovaskainen & Abrego, 2020). One of the advantages of JSDMs is that species are not modelled independently from each other, but it considers both the underlying joint structure related to abiotic filtering (*i.e*., environment) and biotic filtering (*i.e*., species co‐occurrences). The community assembly processes of species communities are a result of multiple processes, including environmental impacts on distribution limits, and the realized species interactions that can favour or disfavour interacting species (Chase, 2003; Leibold *et al*., 2004). Furthermore, the responses of species are determined by species‐specific abilities to cope with environmental change, *e.g.*, the width of niche space and adaptability (Chase & Leibold, 2003) as well as the ability to coexist with other species, *e.g.*, in terms of resource availability and competition (Gravel *et al*., 2011). Therefore, it is crucial to consider these aspects when unravelling community developments, and their yields, under changing conditions.

Recent advances in statistical community models now allow for the integration of abiotic and biotic filtering processes in a spatiotemporal context (Ovaskainen *et al*., 2017; Tikhonov *et al*., 2020). In this study, the authors use a JSDM framework (Ovaskainen & Abrego, 2020) to estimate fishery yield responses in a multispecies complex to environmental variations in the northern Baltic Sea, an area heavily utilized by Finnish fisheries over the past four decades (1980–2018).

Multiple studies have been focusing on the contribution of environmental variations to Baltic Sea fisheries resources in a multispecies context, but those are mostly limited to only a few commercially exploited species, most notably *G. morhua*, *C. harengus* and *S. sprattus* (*e.g.*, Heikinheimo, 2011; MacKenzie & Köster, 2004; Margonski *et al*., 2010). In this study, the authors take advantage of the high‐resolution data of Finnish fisheries on the 16 most exploited species for 48 statistical rectangles (*c*. 50 × 50 km) covering a coastal to offshore gradient as well as the entire latitudinal gradient of Finnish coastal waters. The authors focus on how temporal changes of environmental drivers contribute to the variation in species‐specific fishery yields of a multispecies complex, and model fished yields in response to undergone environmental change over the past four decades as well as habitat characteristics.

## 2 MATERIALS AND METHODS

### 2.1 Research area

**FIGURE 1:**
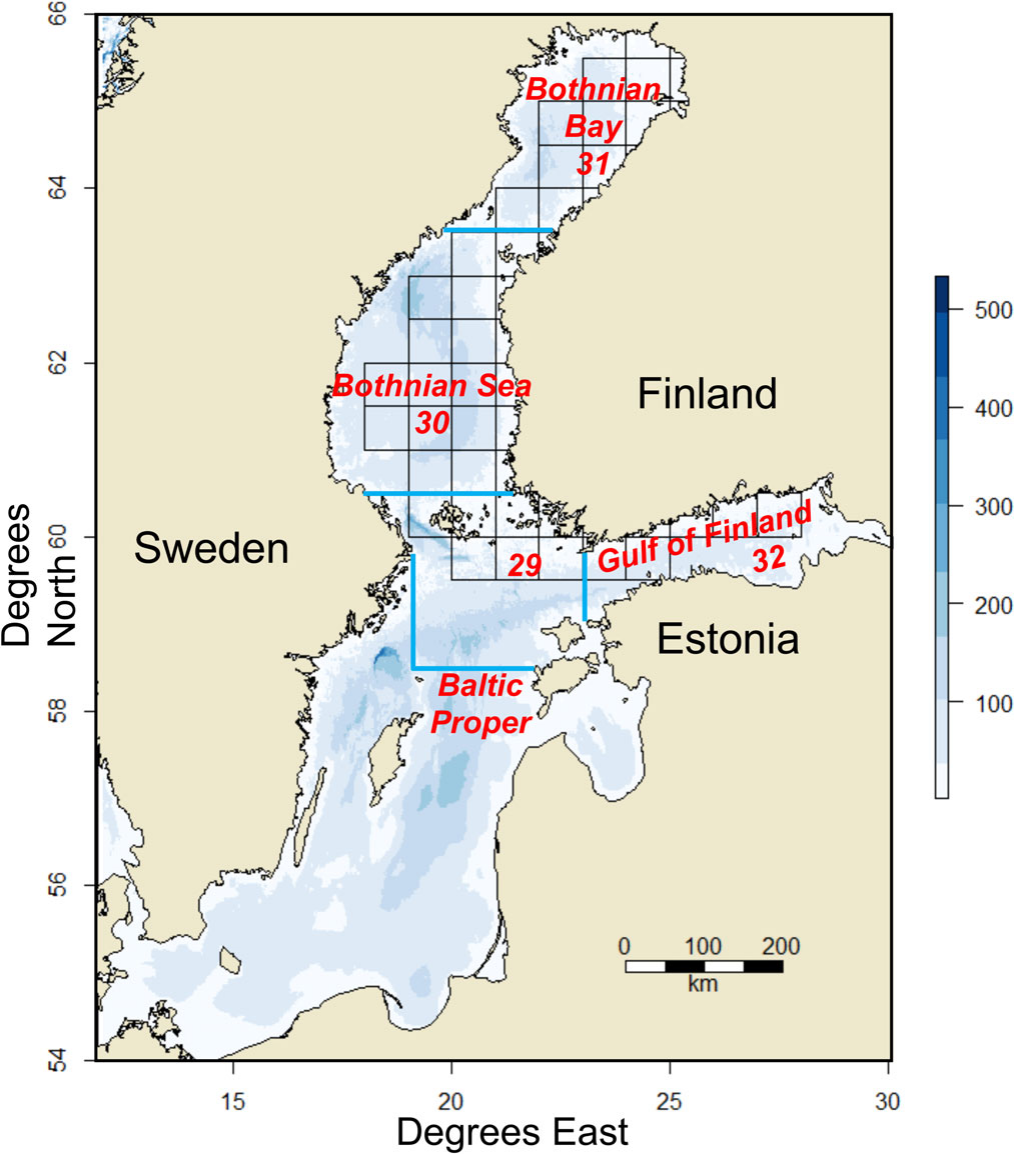
The research area in the Baltic Sea with the statistical grid applied and the ICES subdivisions 29–32 (with blue border line)

The research area encompasses the principal areas utilised by the Finnish fisheries in the Baltic Sea, *i.e*., the Baltic Proper, Bothnian Sea, Bothnian Bay and the Gulf of Finland, conforming to the ICES subdivisions 29, 30, 31 and 32 (Figure 1). The Baltic Sea is a relatively species‐poor non‐tidal semi‐enclosed brackish sea with substantial gradients in several environmental factors, most prominently in salinity, ranging from a maximum of *c*. 30 psu in the westernmost area, the Kattegat, to virtually fresh water in the northernmost and easternmost areas (Snoeijs‐Leijonmalm & Andrén, 2017). The biota consists of a mixture of marine and freshwater species, glacial relicts and recent invaders, many species living close to their tolerance limits regarding one or more environmental factors (Bonsdorff, 2006). For example, low salinity is known to delimit reproduction of *G. morhua* (*e.g.*, Nissling & Westin, 1997), low temperature and salinity delimit the reproduction of *S. sprattus* (MacKenzie & Köster, 2004; Parmanne *et al*., 1994) and low temperature the recruitment of zander (*Sander lucioperca* L.) and perch (*Perca fluviatilis* L.) (Heikinheimo *et al*., 2014; Kokkonen *et al*., 2019). Despite the relatively low species richness compared to fully marine systems, the Baltic Sea is a productive ecosystem supporting major commercial and recreational fisheries (Mackenzie *et al*., 2007). Fished resources in the northern Baltic Sea consist of a unique mixture of marine, anadromous and freshwater species adapted to the brackish water conditions.

The Baltic Sea is a multiple stressed ecosystem due to intensive anthropogenic activities in the drainage area as well as on the sea, with climate change having additionally strong superimposed impacts (Andersson *et al*., 2015; HELCOM, 2018; Reusch *et al*., 2018). The Baltic Sea is warming faster than any other large marine ecosystem (Belkin, 2009). Climate change influences the salinity through climate‐mediated saline water inflow events, precipitation to the sea and the plentiful inflow of fresh water from the extensive drainage area of the sea (BACC II Author Team, 2015; Meier *et al*., 2006). The combined impacts of eutrophication, climate change and other anthropogenic pressures, such as fishing, cause threats to the biodiversity and the integrity of the Baltic Sea ecosystem (Blenckner *et al*., 2021).

### 2.2 Fish data

The fish data consist of commercial yields taken by the different fisheries operating in the research area. Close to the end of the time frame of this study, the fleet consisted of about 1500 active vessels, with the vast majority being coastal fisheries using mainly trap nets, fyke nets and gillnets, whereas the offshore fleet comprised 56 vessels using pelagic trawls (ICES 2000).

Catches‐per‐unit‐of‐effort (CPUE) data have been commonly applied to study the impacts of environmental factors on distribution of fish stocks. Especially for small fish stocks as well as those of lower commercial interest, fisheries‐independent CPUE data are often scarce, not available for different sites and long‐term series and typically reflect a snapshot from a certain site and season, whereas for most fisheries the only available data are the fish biomass caught each year (Carruthers *et al*., 2012; Pauly *et al*., 2013; Martell & Froese, 2013). Nonetheless, it has been shown that trends in catch data are consistent with trends in biomass data of fully assessed stocks, and even maximum sustainable yields could be estimated with great accuracy from time series with catch data (Martell & Froese, 2013).

The spatial distribution of most fish species in the Baltic Sea is poorly known. Even for the handful of species which are within the formal fish stock assessment (ICES 2020), the distribution data are typically restricted to a single annual survey excluding the coastal areas and the northernmost Baltic Sea. In addition, these surveys cover only short time series.

Coastal fish community monitoring with gillnets has been conducted in a few scattered locations in the Baltic Sea (*e.g.*, Bergström *et al*., 2016; HELCOM, 2012; Olsson *et al*., 2012).

Considering the utility of CPUE data, it is well known that even for a single gear type several environmental factors, such as water clarity, may substantially influence the catchability (Olin *et al*., 2016). Furthermore, due to species‐ and size‐dependent factors, as well as to changes in fish behaviour and catchability, gillnet catches never reflect a true image of the abundance of a fish species or the entire fish community, but are a sample of the catchable assemblage considering the used gillnet (Mustamäki & Mattila, 2015; Olin *et al*., 2016).

Even if CPUE data were available from commercial fisheries, there are inconsistencies – *e.g.*, the trawl sizes in Finnish northern Baltic Sea herring fishery nearly tripled during 20 years, influencing the catchability of fish and thus, the utility of CPUE data (Rahikainen & Kuikka, 2002). In addition, large variations in the growth rates of herring in the northern Baltic Sea have influenced the selectivity of trawls (Rahikainen *et al*., 2004). Such inconsistencies in CPUE data may pose challenges to account for when assessing environmental impacts on fisheries resources. Considering the small and scattered coastal fish stocks in this study, standardization of fishing effort data is impossible as multiple gear types with spatial and temporal variations have been used in a research area encompassing large variations and trends in several environmental factors.

Because of the scarcity of CPUE data from monitoring and commercial‐fisheries, and considering a multispecies, multiple gear, decades‐long and spatially heterogeneous environment, the authors take advantage of detailed data stemming from commercial fisheries in Finland. They treat the yield data as the best available proxy, reflecting the variations in the commercial yield resources of the included species, enabling us to use this expectational data set, while specifically accounting for varying annual efforts in the statistical framework of this study (see below). The species‐specific total yield data of the Finnish fisheries were compiled from commercial fisheries covering the years 1980–2018. Altogether, a total of 16 of the most important species, indicated by yields, were included in the analysis of this study, whereas species with low and sporadic yields were not included in the analyses (Table 1).

**TABLE 1:** Overview of included fish species, their taxonomic grouping, common name, origin including temperature affinity (HELCOM, 2012) and average annual total yields with standard deviation (s.d.) during 1980–2018

Common name	Scientific name	Family	Short name	Origin	Annual total yields (t)	
					Average	s.d.
Atlantic herring	*Clupea harengus* L.	Clupeidae	Herring	Marine, cold	82,345	16,734
European sprat	*Sprattus sprattus* L.	Clupeidae	Sprat	Marine, cold	7458	6334
Freshwater bream	*Abramis brama* L	Cyprinidae	Bream	Freshwater, warm	295	239
Ide	*Leuciscus idus* L.	Cyprinidae	Ide	Freshwater, warm	20	8
Roach	*Rutilus rutilus* L.	Cyprinidae	Roach	Freshwater, warm	216	164
Northern pike	*Esox lucius* L.	Esocidae	Pike	Freshwater, warm	212	33
Atlantic cod	*Gadus morhua* L.	Gadidae	Cod	Marine, cold	818	1395
Burbot	*Lota lota* L.	Lotidae	Burbot	Freshwater, cold	96	45
European smelt	*Osmerus eperlanus* L.	Osmeridae	Smelt	Freshwater, warm	463	311
European perch	*Perca fluviatilis* L.	Percidae	Perch	Freshwater, warm	618	256
Zander	*Sander lucioperca* L.	Percidae	Zander	Freshwater, warm	353	157
European flounder	*Platichthys flesus*. (L. 1758)	Pleuronectidae	Flounder	Marine, cold	40	29
Vendace	*Coregonus albula* L.	Salmonidae	Vendace	Freshwater, cold	141	71
Maraena whitefish	*Coregonus maraena* Bloch 1779	Salmonidae	Whitefish	Estuarine/anadromous, cold	918	283
Atlantic salmon	*S. salar* L.	Salmonidae	Salmon	Anadromous, cold	535	454
Sea trout	*Salmo trutta* L.	Salmonidae	Sea trout	Anadromous, cold	96	69

The annual summarized commercial yields, based on obligatory reporting by commercial fishers, were available from the ICES statistical rectangles with dimensions 0.5 latitude degree × 1 longitude degree (Statistics Finland, http://pxnet2.stat.fi/PXWeb/pxweb/en/StatFin/StatFin__maa__akmer/statfin_akmer_pxt_12d5.px).

### 2.3 Environmental data

The environmental data cover the same geographic area and time frame as the yield data, including information on physico‐chemical water characteristic and the topography in each statistical rectangle (Table 2). The topographic data consider rectangle‐specific values of shore density, a measure that has previously emerged as an important predictor of fish production (Tolvanen *et al*., 2004; Uusitalo *et al*., 2012). Shore density is the ratio of coastline length (km) to water area (km^2^) in each rectangle, thus being static over time. The Finnish shoreline largely consists of complex mosaic‐like structures of land and sea while there are also open areas towards the sea. The authors assume that a long shoreline facilitates fish production, because shallow near‐shore areas provide essential habitats for fish for reproduction, feeding and growth to maturity (Kraufvelin *et al*., 2018; Sundblad & Bergström, 2014).

**TABLE 2:** Water quality and topography variables, units, water layer and season from which the samples were included, as well as the annual minimum, mean and maximum number of rectangles sampled for the different environmental variables during 1980–2018

Variable	Unit	Water layer	Season	Number of annually sampled rectangles		
				Minimum	Mean	Maximum
Total nitrogen concentration	mg m^3^	0–5 m	July–September	29	36	43
Oxygen concentration	mg l^−1^	Largest depth sampled, 1–5 m above bottom	July–September	29	36	43
Chlorophyll‐*a* concentration	mg m^3^	2*Secchi depth	July–September	9	20	26
Temperature	°C	0–5 m	July–September	29	36	43
Salinity	‰, PSU	0–5 m	July–September	29	36	43
Shore density	Unitless ration: shoreline (km)/water area (km^2^)	—	Static value	—	—	—

The water quality data encompass physical, chemical and biological variables, and rely on the information from extensive Finnish monitoring programmes (open access interfaces for environmental data – syke.fi). The data were compiled from water layers and seasons considered appropriate for each variable in the current study (Table 2). Data from summer were selected as this is the most important season for biological processes (Möllmann *et al*., 2004; Viherluoto *et al*., 2000; Viitasalo *et al*., 1995). Each of the 48 statistical rectangles included on average *c*. 21 sampling stations and 240 yearly measurements resulting in a total of *c*. 11,600 environmental observations per year. Overall, the proportion of missing values in rectangle‐specific data was on average 31.5%, and this fraction was filled in by spatial interpolation (Table 2).

Salinity and temperature are known to influence fish stocks as exemplified above. Besides, in the Baltic Sea, eutrophication has shown to increase fish production over cascading effects from lower trophic levels (Eero *et al*., 2016; Elmgren, 1989; Thurow, 1997). As the authors do not have detailed spatiotemporal information on the distribution of various food resources for fish, such as phytoplankton, zooplankton, zoobenthos or forage fish, they used total nitrogen and chlorophyll *a* concentration as proxy for ecosystem productivity. Total phosphorus was also considered as a potential variable to contribute to productivity of the ecosystem and fish stocks, but it was rejected because of strong correlations with other covariates.

With current operational physical‐biogeochemical models not being able to reliably model nutrient cycling, salinity and temperature in the Gulf of Bothnia (Eilola *et al*., 2011; Fransner *et al*., 2018; Savchuk *et al*., 2012), which comprises most of the research area of this study, the authors refrained from using a biogeochemical hydrographic climate model, and instead used the high quality time‐series data and spatial interpolations to best capture the environmental variability in the study area.

To increase the spatiotemporal resolution of environmental covariates in cases of missing values in the statistical rectangles, and to include all available fish community data, the authors applied spatial interpolation imputation. This is a common procedure for filling gaps in water quality elements at locations where they have not been measured (Murphy *et al*., 2010) or to complete time series (di Piazza *et al*., 2011). Inverse distance weighting (IDW) from the R‐package “gstat” (Pebesma 2004, Gräler *et al*. 2016) was applied in spatial interpolation for annual data of each water quality descriptor, and subsequently, annual rectangle‐specific averages were calculated from the resulting surface.

Fisheries in the Baltic Sea exploit cohorts of fish born several years earlier. Thus, the environmental covariates have been influencing the harvested cohorts already during multiple years. Time‐lags between environmental change and population response are well known in ecology (Watts *et al*., 2020) and in fisheries research (Probst *et al*., 2012). As the authors model a multispecies complex simultaneously, and thus cannot apply species‐specific moving averages, based on, *e.g.*, age at maturity, they applied a conservative moving average for all species following previous studies in the study area (Snickars *et al*., 2015; Uusitalo *et al*., 2012). Therefore, the authors calculated 5 year moving averages of the environmental variables based on the data from the spatial interpolations including the current and four preceding years. Being aware of the potential movement of fish between spatial units, previous research from the study area of this study suggests that the movement between spatial rectangles can be neglected in a fish community study (Uusitalo *et al*., 2012).

### 2.4 Statistical analysis

Because the aim of this study was to model the responses of multiple fished species’ yields to environmental covariates simultaneously, the authors used a JSDM, the Hierarchical Modelling of Species Communities (Hmsc) (Ovaskainen *et al*., 2017; Tikhonov *et al*., 2020) from the “Hmsc” R package (Ovaskainen & Abrego, 2020; Tikhonov *et al*., 2019). Hmsc allowed the authors to model the correlations between species‐specific responses to the environmental covariates. The response variables of this study were the fishery yields (kg km^−2^) of 16 species at each of the 48 statistical rectangles between 1980 and 2018, that the authors log‐transformed [ln (yield +1)] before the analysis.

The authors included surface water temperature, salinity, total nitrogen concentration, near‐bottom water oxygen concentration, chlorophyll *a* concentration and shore density as fixed effects. Because the species typically have a certain optimum in their environmental niche, the authors modelled the associations with water quality covariates also accounting for second‐order polynomial responses, whereas the shore density was included as a linear predictor. To account for underlying temporal trends not described by included covariates, the authors also included year as a linear fixed effect. To further account for the temporal stochasticity of the data, *i.e*., the unstructured variation of data at the year level, also comprising the varying fishing effort over time, the authors included year also as temporal random effect.

The authors fitted the models using the Markov Chain Monte Carlo (MCMC) method implemented in Hmsc and examined all simulations and convergence with the Gelman‐Rubin Potential Scale Reduction Factor (Gelman & Rubin, 1992). Subsequently, they evaluated the model fit and summarized the overall explanatory power as the community mean *R* ^2^ across all species‐specific *R* ^2^. They then used a fivefold cross‐validation procedure to assess the predictive power of the model. Finally, they explored the parameter fits and partitioned the species‐specific explained variation to the environmental covariates following Ovaskainen *et al*. (2017).

## 3 RESULTS

### 3.1 Model evaluation

The MCMC convergence of this study's model was satisfactory, indicated by the potential scale reduction factor for the beta‐parameters (response of yields to environmental covariates *sensu* Ovaskainen *et al*., 2017) being <1.1 (average 1.003, mean upper c.i. = 1.012). This study's model showed a good fit with a mean *R* ^2^ value of 0.56 for explanatory power, and a predictive *R* ^2^ value of 0.54 resulting from a five‐fold cross‐validation procedure.

### 3.2 Environmental impact on species yields

**FIGURE 2:**
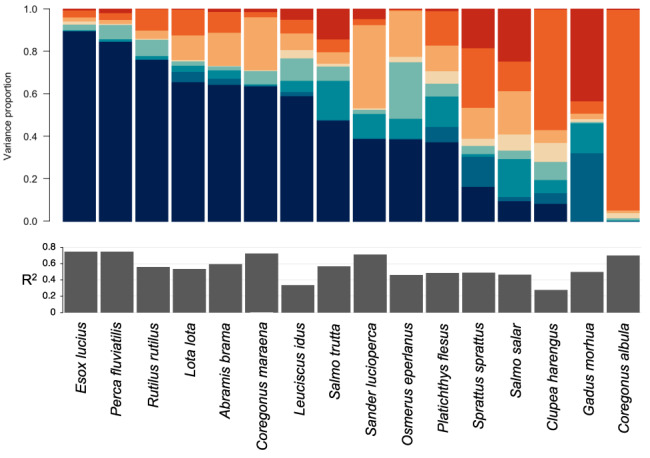
Results of variance partitioning. Variation in species yields is partitioned into responses to fixed and random effects. The upper bar‐plot shows species‐specific results whereas the legend shows, in addition to the colour scales, the averages over all species. The lower bar‐plot indicates the species‐specific *R* ^2^ values. Species are sorted from left to right following decreasing influence of shore density. 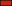 Random:Year (mean = 7.5); 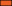 Salinity (mean = 16.9); 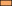 Bottom oxygen (mean = 11.9); 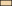 Chlorophyll *a* (mean = 2.6); 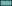 Total nitrogen (mean = 6); 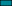 Temperature (mean = 6.8); 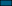 Year (mean = 4.5); 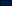
Shore density (mean = 43.7)

The explained variation was highest (≥70%) for the yields of *P. fluviatilis*, pike (*Esox lucius*), *S. lucioperca* and the two coregonids, vendace (*Coregonus albula*) and whitefish (*Coregonus maraena*) (Figure 2). The authors found higher explained variation in coastal freshwater species compared to migratory ones, most notably salmon (*Salmo salar*), and marine species, in particular *G. morhua* and *C. harengus*.

Using the JSDM approach highlighted the diverging responses of species‐specific yields to the environmental factors (Figure 2). Shore density and salinity contributed most to the explained variation, explaining on average of 43.7% and 16.9%, respectively (Figure 2). Shore density was particularly important for the freshwater species, but *C. albula* was a clear exception with negligible association with shore density (Figure 2). *G. morhua* had only weak association with shore density and *S. sprattus* was the only species showing a negative response (Figure 4).

**FIGURE 3:**
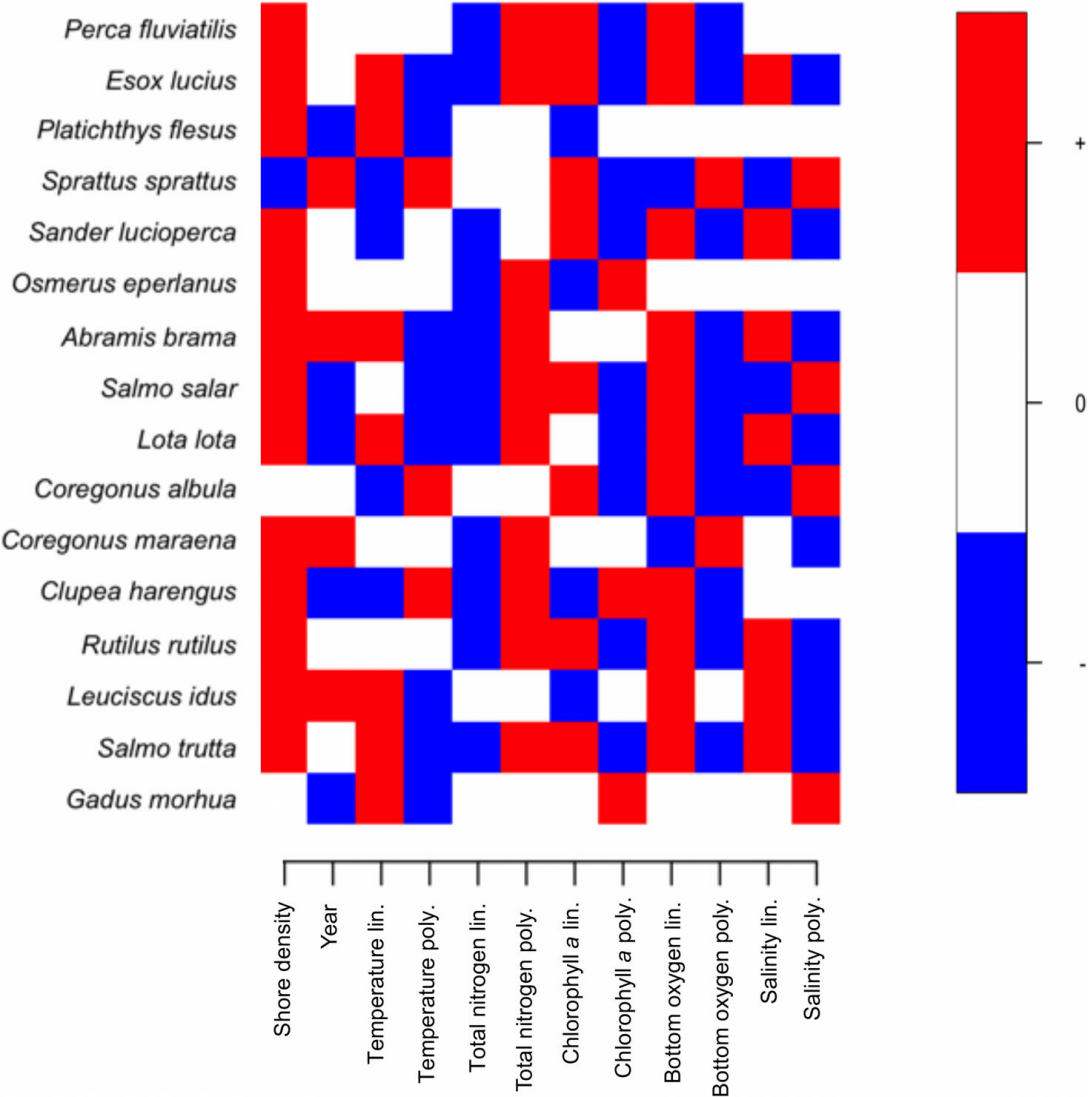
Species yield–environment responses with support of at least 95% posterior probability. Red indicates positive and blue negative correlations with the environmental variables including both the linear and polynomial components

Salinity was the strongest driver for *C. albula*, *C. harengus* and *S. sprattus* (Figure 3). For *C. albula* other covariates only had marginal contributions. Near‐bottom oxygen concentration emerged as the second strongest water quality covariate and contributed particularly to the explained variation of *C. maraena*, *S. lucioperca*, smelt (*Osmerus eperlanus*) and *S. salar*.

On average, temperature contributed to almost 7% to the explained variation. *G. morhua*, flounder (*Platichthys flesus*) and *S. salar* showed highest proportions of explained variation by temperature, with all three showing strong negative responses to the quadratic function of temperature (Figures 2 and 3). Covariates serving as proxy for ecosystem productivity, *i.e.*, total nitrogen and chlorophyll *a* concentrations, contributed most to the variation in *S. lucioperca*, *C. maraena*, *O. eperlanus* and *S. salar* yields (Figure 2).

**FIGURE 4:**
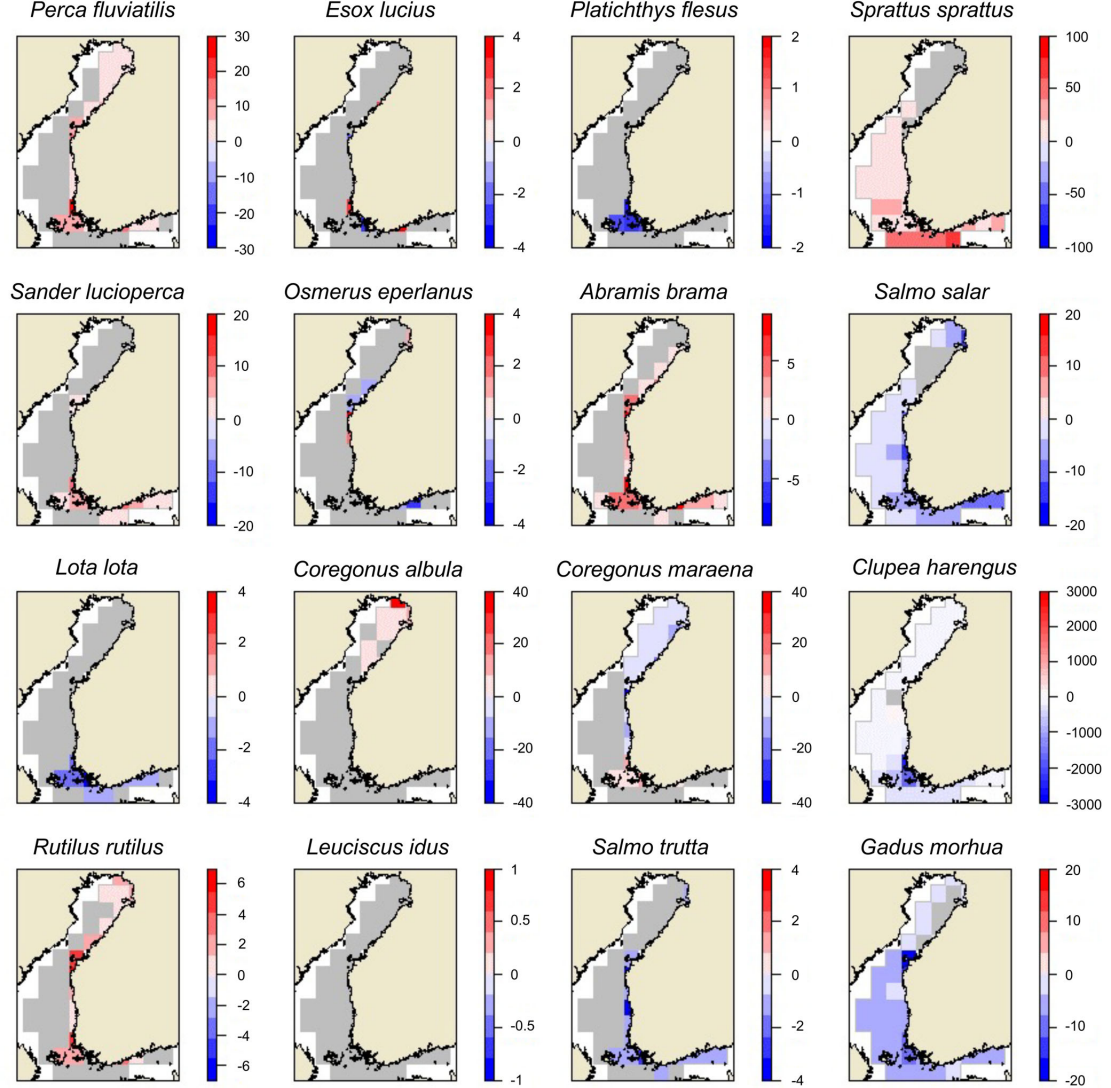
Differences in modelled annual average yields (kg km^−2^) in 2010–2018 compared to the yields in 1984–1992 (= yields in 2010–2018 − yields in 1980–1992). In the grey rectangles the predicted changes between the two periods were <1 kg km^−2^. Note the species‐specific legend scales

Following the environmental progression, the results of this study point to considerable spatiotemporal changes in the species‐specific yield. This is evident by comparing the differences between decadal time frames covering roughly the 1980s (1984–1992) and 2010s (2010–2018) (Figure 4).

**FIGURE 5:**
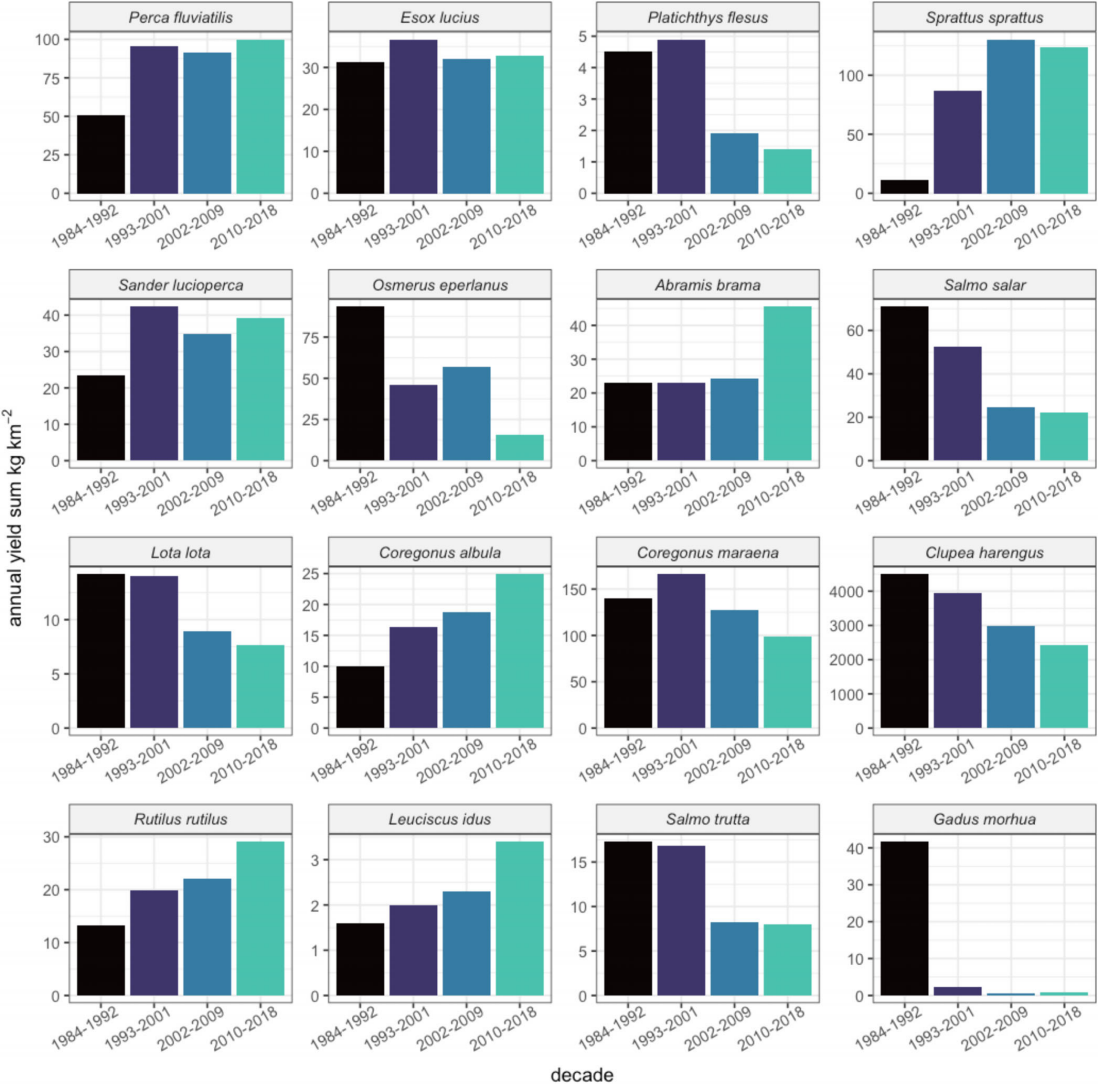
Predicted decadal sums of species‐specific commercial yields (kg km^−2^)

This study's model suggests environment‐driven increases in *P. fluviatilis* yields in all coastal areas and increases in *S. lucioperca* in the southern coastal areas. Total *E. lucius* yields remain relatively stable over time (Figure 5) with some specific alternating trends in space (Figure 4). The model pointed to strong decreases in the yields of *G. morhua* and *P. flesus* for their whole distribution area over time. *C. harengus* yields were predicted to decrease in the south‐western area, the Archipelago Sea, with little changes in other sea areas. In contrast, the results of this study predicted substantial environment‐driven increases in sprat yields over time in these areas (Figure 4). The model further highlights an overall decrease in yields for *S. salar* and sea trout (*Salmo trutta*) in relation to environmental change. The authors found increasing yields for bream (*Abramis brama*) and roach (*Rutilus rutilus*) in virtually all rectangles close to the coast. The decrease in yields for burbot (*Lota lota*) was strongest in the south. Increasing *C. albula* yields were predicted in the northernmost part of the Baltic Sea. The predicted *C. maraena* catches peaked in 1993–2001 but decreased thereafter in the northern and increased in the south‐western archipelago areas (Figure 5).

## 4 DISCUSSION

The production and distribution of fish species as well as fisheries resources are largely governed by environmental conditions and climate variability (Brander, 2007; Castillo *et al*., 1996; Perry *et al*., 2005; Pörtner & Knust, 2007). In this study, the authors demonstrate that a substantial fraction of the variations in fishery yields can be attributed to environmental changes. Following progressing climate change, it is likely that the impacts of environmental factors will have an even stronger effect on fishery resource dynamics in the future. In the Baltic Sea, the fish community comprises freshwater, anadromous and marine species with both warm and cold‐affinity species. Despite species‐specific responses to environmental change over the past four decades, the results of this study highlight that especially warm‐affinity freshwater species are likely to benefit from ongoing environmental change in the Baltic Sea, leading to higher yields, whereas most cold‐affinity marine or anadromous fish species showed decreasing yields. Nonetheless, the modelled positive development of *S. sprattus* could be seen as an exception to the observed pattern as it is here considered a marine cold‐water species (HELCOM, 2012). Yet, there is evidence for gradually warming conditions being favourable for the egg survival and recruitment success of *S. sprattus* (Petereit *et al*., 2008; Voss *et al*., 2011), also supporting the findings of this study. Besides the environmental impact on *S. sprattus*, the positive development may also be supported by released predation pressure, especially from *G. morhua* (Parmanne *et al*., 1994; Tomczak *et al*., 2022).

The results of this study, based on 16 fished species, strengthen previously established conclusions suggesting that marine species in the Baltic Sea are disfavoured with contracting habitats, which gradually become warmer and less saline, whereas freshwater habitat expansion would benefit freshwater species (Mackenzie *et al*., 2007). Such developments have been anticipated following future climate projections until the end of the 21st century (Mackenzie *et al*., 2007). Nonetheless, the authors of this study show that the diverging success of marine and freshwater species and their resulting yields in response to environmental change became already apparent during the past decades.

The explained variation by environmental factors was in general higher for coastal freshwater species than in marine species (Figure 2). This may be because of larger areal movements of marine and anadromous species, where local environmental variation contributes less to the dynamics of fishery resources. Freshwater species, on the contrary, typically spend their whole life history in relatively limited area (*e.g.*, Uusitalo *et al*., 2012). Nonetheless, compared to other recent studies linking catches or CPUE to environmental variation, the model of this study was able to capture considerably higher explained variation, indicated by the *R* ^2^ values (*e.g.*, Olsson *et al*., 2012; Orio *et al*., 2020; Parra *et al*., 2017).

The large contribution of shore density compared to water quality descriptors suggests that the environmental conditions were widely in the tolerance ranges of several freshwater species. Moreover, the modelling results of this study support that the environment became more favourable for warm‐affinity freshwater species, above all percids and cyprinids, as the potential fishery yields of percids and cyprinids are predicted to increase in wide areas. This development can be attributed to the consistent warming of the Baltic Sea favouring warm water species (Ådjers *et al*., 2006; Olsson *et al*., 2012; Snickars *et al*., 2015).

Considering the marine species, it becomes clear that the environmental regime was more favourable for, *e.g*., *P. flesus* and *G. morhua* in the early years of this study, but changes in multiple factors contributed to the drastic decrease in the resources these species provided to fisheries. The findings of this study concerning *P. flesus* are in line with Jokinen *et al*. (2015, 2016), reflecting that unfavourable changes in salinity during critical phases affect reproduction, as well as habitat change and loss due to eutrophication affect survival and distribution. Likewise, it is apparent that the eastern Baltic *G. morhua* stock has suffered from unfavourable environmental conditions including eutrophication, hypoxia in deep water and decreasing salinity (Hinrichsen *et al*., 2011; Köster *et al*., 2005; Limburg & Casini, 2018; Vallin *et al*., 1999).

For *C. harengus*, the most important species for the Finnish commercial fishery, indicated by landings and the value of landings (Natural Resource Institute Finland (Luke), 2020), the model of this study showed decreasing yields towards the past decade of this study. This supports that environmental conditions may become less favourable for this marine species, although it must be noted, that in this model the explained variation was lowest for herring. In the Gulf of Finland, chlorophyll *a* concentrations exceed the optimum level for *C. harengus* recruitment as a result of eutrophication, and decreasing salinity further contributes to unfavourable environmental conditions for *C. harengus* (Rahikainen *et al*., 2017). In contrast, the authors show that environmental changes seem to strongly favour *S. sprattus* yields.

This model's results underpin largest increases in *S. sprattus* in the south‐western areas but also increasing yields in the Bothnian Sea. The expansion of *S. sprattus* northwards is, nonetheless, delimited by its reproduction as the survival of eggs decreases below the salinity of 8 psu and below 5 psu the development of eggs is not completed (Petereit *et al*., 2009).

Metapopulation structure and source‐sink dynamics may pose some challenges when seeking to unravel the contribution of environmental factors on the dynamics of mobile fish species, as local population dynamics may depend on migrations of specimens from more productive areas (Casini *et al*., 2012; Lindegren *et al*., 2014). As suggested in the case of *C. harengus* (Mariani *et al*., 2005; McPherson *et al*., 2003; McQuinn, 1997) dynamics and population structure can be described with the theory of metapopulations, displaying a certain degree of isolation between spawning populations, but showing some seasonal dispersal during feeding. This is supported by persisting, although weak, genetic structuring among spawning populations in the Baltic Sea (Teacher *et al*., 2013). It is thus possible that the specimens caught from a certain area have not experienced similar environmental variations throughout their whole lifetime, which could contribute to the relatively smaller explained variation (*R* ^2^ values) by the local environment the authors found for partly migratory species in this study. In the case of *G. morhua*, the reproductive surpluses from spawning areas in the Baltic Proper, especially during high population levels, maintain populations in basins where environmental conditions are unfavourable for reproduction (Casini *et al*., 2012). Considering environmental changes in remote, non‐surveyed areas could be one of the issues to consider in future work.

Studies estimating the associations between environmental factors and abundances of multiple fish species (or proxies of abundances such as CPUE or total catch) in the Baltic Sea have applied a wide range of analytical methods, depending on the aims of the study (*e.g*., Juntunen *et al*., 2012; Olsson *et al*., 2012; Peltonen *et al*., 2007; Uusitalo *et al*., 2012). The current study is to the best of the authors’ knowledge the first one to apply a JSDM approach, which provides virtues over many other modelling approaches as it also accounts for species associations on a community level and enables the prediction of the responses of all species and their associations with changes in multiple environmental factors simultaneously.

The results of this study revealing associations of fishery yields with environmental covariates show that despite species‐specificity, salinity, bottom oxygen concentration and temperature were the most important covariates in explaining the yields of 16 fished species. This is partly in contrast with findings from Uusitalo *et al*. (2012) describing the effects of salinity to be negligible. Nonetheless, in line with the current study, they showed that yields of almost all species were lowest at low shore densities. Several species had bell‐shaped response to chlorophyll *a* suggesting that increasing eutrophication first supports the fishery resources these species provide but excessive eutrophication would have negative impacts. The same holds true for the responses to deep‐water oxygen (Figure 3).

Olsson *et al*. (2012) showed that long‐term changes in the composition of catches in coastal fish monitoring were mainly structured by climate‐related environmental changes such as temperature, salinity and the North Atlantic Oscillation index, and less by nutrient concentrations, supporting the findings of this study. Nonetheless, they did not include species‐specific responses to environmental covariates, nor topographic characteristics, which the authors found fundamental in this study. The use of covariates reflecting geographic and topographic aspects has proven beneficial in explaining coastal and offshore fish distributions, often having a higher explanatory power than hydrographic covariates (Juntunen *et al*., 2012; Peltonen *et al*., 2007), which is also supported by the results of this study.

Climate change is likely to induce a major redistribution of global marine catches (Cheung *et al*., 2009). These projections also included the Baltic Sea, pointing to increasing fishery resources in the Bothnian Sea and Bothnian Bay until 2055, partly relying on projections of primary production. Indeed, during the 20th century, eutrophication was one of the key factors to contribute to the *c*. 10‐fold increase in fish catches in the Baltic Sea (Elmgren, 1989; Thurow, 1997). Moreover, following the trends in environmental parameters suggests that primary production (indicated by chlorophyll *a* concentrations) is increasing in the research area of this study, but also that oxygen concentrations are largely decreasing. Fisheries yields may not increase with increasing eutrophication if declining oxygen concentrations decrease habitat quality further.

The current study highlights the utility of JSDM frameworks to better understand the responses of fished resources to environmental variations. The modelling indicated, *e.g*., that in many coastal areas, environmental trends are likely to be harmful for *S. salar* and *S. trutta*, as well as for *L. lota*. The demersal marine fish species *P. flesus* and *G. morhua* are threatened in this area because of the ongoing environmental changes, even if excessive exploitation of *G. morhua* during a long period (*e.g.*, Eero *et al*., 2011, 2020) has had a major contribution to the current poor status in the northern Baltic Sea. The environmental changes in the Baltic Sea are rapid, whereby the species are not able to adapt, and in this semi‐enclosed sea, the niches released by disappearing marine species are difficult to occupy by migrators from conspecific populations outside the Baltic Sea especially as survival in the Baltic Sea may demand specific genetic adaptations (Johannesson *et al*., 2011).

The framework of this study can support current ecosystem based‐management tools by evaluating spatiotemporal predictions of species in a community context while accounting for environmental change. The usefulness of linking modelling frameworks for management purposes has been shown in cases where species distribution modelling supports spatiotemporal food‐web models, such as *Ecospace* (Coll *et al*., 2019). Alternatively, as *Ecospace* applies environmental envelope modelling for species distributions (Christensen *et al*., 2014), JSDM could provide support for *Ecospace* models through provisioning the forcing of species when considering their environmental preferences. Likewise, considering end‐to‐end ecosystem models such as the *Atlantis* for which a Baltic Sea implementation has been developed (Bossier *et al*., 2018), JSDM could be applied for model verification or to force the spatial distribution of fish, as they better account for species dependencies in a community complex than solely considering environmental envelopes.

Nonetheless, even if learning about community‐wide responses of fish to environmental changes and novel modelling approaches could support sustainable management of natural resources, *e.g*., in the northern Baltic Sea, the ongoing rapid environmental changes induced by humans constitute a severe threat for the locally adapted fish species and the resources they provide.

## FUNDING INFORMATION

This research was funded by the Strategic Research Council of the Academy of Finland (grant 312650 to the BlueAdapt consortium).

## CONFLICTS OF INTEREST

The authors declare that they have no known competing financial interests or personal relationships that could have appeared to influence the work reported in this paper.

## Data Availability

The data underlying this article are available in the Statistics Finland PxWeb database the open access interface for environmental data provided by the Finnish Environment Institute (SYKE), and the Natural Resource Institute Finland (Luke) statistical services. The data sets were derived from sources in the public domains:
http://pxnet2.stat.fi/PXWeb/pxweb/en/StatFin/StatFin__maa__akmer/statfin_akmer_pxt_12d5.px for fish data
https://www.syke.fi/en-US/Open_information/Open_web_services/Environmental_data_API#WQ for environmental covariate data
